# Role of aurora kinase B in regulating resistance to paclitaxel in breast cancer cells

**DOI:** 10.1007/s13577-022-00675-8

**Published:** 2022-01-28

**Authors:** Min Liu, Yinan Li, Cui Zhang, Qing Zhang

**Affiliations:** 1 Department of Oncology, Beijing Hospital of Traditional Chinese Medicine, Capital Medical University, No. 23 Art museum Back street, Dongcheng District, Beijing, 100010 China; 2 Department of Oncology, Beijing Hospital of Traditional Chinese Medicine, Capital Medical University, Beijing, 100010 China; 3 Graduate School of Beijing University of Chinese Medicine, North Third Ring East Road 15, Chaoyang District, Beijing, 100029 China

**Keywords:** AURKB, Phosphorylation, Breast cancer, Drug resistance, Paclitaxel

## Abstract

Aurora kinase B (AURKB) is a type of functional kinase with primary functions of participating in cell mitosis, which has been identified to be involved in the occurrence and development of malignant tumors strongly. However, it still remains a controversial with respect to the relationship between the phosphorylation level of AURKB and its function. In our initial research, there was no significant difference in the relative content of AURKB protein between drug-resistant breast cancer cells and wild-type cells; however, its phosphorylation level in drug-resistant cells was significantly higher than that in wild-type cells. Subsequent cell and animal experiments both confirmed the positive correlation between AURKB phosphorylation and drug resistance. Furthermore, PRKCE in the upstream was identified to regulate the phosphorylation of AURKB, which promoted the change of spatial localization of AURKB from nucleus to cytoplasm. Accordingly, phosphorylated AURKB reduced the negative regulation of downstream RAB27B transcription physically, and interacted with RAB27B in cytoplasm to maintain its protein stability. Eventually, it promoted exosome secretion of drug-resistant cells and drug efflux. Using shRNA to knockdown AURKB expression, using hesperadin to inhibit AURKB activity, mutating the AURKB phosphorylation site, or using siRNA as well as BIM to inhibit the activity of the upstream AURKB phosphorylation regulatory protein PRKCE, all of which directly or indirectly reduce AURKB phosphorylation, are effective in reversing PTX resistance in cells. Collectively, this study provides experimental evidence for PRKCE/AURKB/RAB27B axis in regulating the resistance to paclitaxel (PTX) in breast cancer cells, offering a potential intervention target for reversing drug resistance.

**Supplementary Information:**

The online version contains supplementary material available at 10.1007/s13577-022-00675-8.

## Introduction

Aurora kinases, which were first discovered in *Drosophila melanogaster*, are serine/threonine kinases that play important roles in cell cycle regulation [1]. This family is named Aurora since mutation of its members causes the centrosome to be unable to separate, leading to the formation of monopolar spindles. Since their discovery, Aurora kinases have been reported in different species. For instance, a single Aurora kinase was found in yeast; Aurora-A and Aurora-B kinases were reported in *Caenorhabditis elegans*, *Drosophila melanogaster* and Xenopus; and mammals have Aurora-A, Aurora-B and Aurora-C kinases [2–5].

Aurora kinase B (AURKB) is a functional kinase located in the centrosome of the nucleus. Its main role is to participate in cell mitosis, and it is mainly located in the middle of the spindle during cytoplasmic division [6]. Recent studies speculated that its functions in mitosis include (1) chromosome condensation; (2) formation of bipolar spindles; (3) chromosome attachment in the mitotic spindle; (4) adjustment of the spindle checkpoint; and (5) completion of cytoplasmic division [7, 8]. Chromosome condensation is the basis of DNA separation and replication. Yasui et al. found that phosphorylation is an important way to activate the activity of AURKB [9]. During the process of chromosome condensation and cell division, AURKB requires histone H3 phosphorylation to promote the condensation of mitotic chromosomes. On the other hand, AURKB can regulate mitotic centromere-associated kinesin (MCAK) via phosphorylation [6, 10]. MCAK is part of a group of kinesins that are associated with mitotic centromere movement [11]. It can bind centromeres in a targeted manner, regulate microtubules, properly aggregate chromosomes, and correct improper centromere-microtubule attachment. According to several previous studies, changes in the expression of AURKB may lead to the failure of cell mitosis, resulting in the production of polyploids [12], which is also considered a prerequisite for the occurrence of malignant tumors.

In recent decades, AURKB has become a hot spot in cancer research [13, 14]. To date, it has been reported that AURKB is highly expressed in various malignant tumors, including colon adenocarcinoma, thyroid follicular carcinoma, laryngeal carcinoma, lung cancer, etc. However, it was discovered that AURKB is not phosphorylated in many tumor tissues [15, 16]. In these malignant phenotypes, AURKB is overexpressed in an inactive state. AURKB phosphorylation is not necessary during the generation of multinucleated and polyploid tumor cells [14, 17, 18].

As revealed in our previous experiment, there was no significant difference in the relative expression of AURKB in breast cancer cells (MDA-MB-231 and BT549) and the corresponding PTX-resistant cells (MDA-MB-231/PTX and BT549/PTX). However, the phosphorylation level of AURKB in drug-resistant cells was significantly higher than that in wild-type cells. Therefore, the present study was performed to explore the relationship between AURKB phosphorylation and resistance to PTX to expand the understanding of the roles of AURKB in tumor cells.

## Materials and methods

### Tissue samples

This study included 12 patients with triple-negative breast cancer who were treated at the Department of Oncology of Beijing Hospital of Traditional Chinese Medicine from January 2018 to March 2021. The patients were diagnosed with invasive ductal carcinoma of the breast by paraffin pathology, and immuno-histochemical examination showed hormone receptor negativity (ER < 1% and PR < 1%), and HER-2 staining was negative or 1+. The average age of the patients was 61.8 years (17–89 years). Patients were subjected to puncture under the guidance of preoperative ultrasound to collect specimens or remove samples during the operation. Collection of the tissue specimens were approved by the Ethics Committee of Beijing Hospital of Traditional Chinese Medicine (No. 2021BL02-006-03), and the patients signed informed consent forms before operation.

### Immunohistochemistry (IHC)

The tissue sections were examined by H&E staining for histological verification of disease status. Sections were dewaxed in xylene and rehydrated in fractionated ethanol. Heat-induced antigen retrieval was performed for epitope unmasking in 10 mM citric acid buffer (pH 6). Slides were blocked with 2.5% horse serum at room temperature (RT) for 1 h and incubated with primary antibody overnight in a humidified chamber at 4 °C. Next, the slices were incubated overnight at 4 °C in primary antibody solution (anti-AURKB, 1:500, ab45145; Abcam, Cambridge, MA, USA). After repeated washing, the slices were incubated with the secondary antibody (peroxidase-conjugated Affinipure goat anti-mouse IgG (H + L), 1:200 dilution, ZB-2305, ZSGB-BIO, Beijing, China) for 1 h at room temperature. The slides were then incubated with 3,3′-diaminobenzidine (DAB) and counterstained with hematoxylin. Image acquisition was performed with an Olympus IX71 fluorescence microscope.

### Cell culture and transfection

Human non-triple-negative breast cancer cell lines (MCF-7 and ZR-75-1) were provided by the Cell Resource Center, Peking Union Medical College. Triple-negative breast cancer cell lines (MDA-MB-231 and BT549) and corresponding Taxol-resistant cell lines (MDA-MB-231/PTX and BT549/PTX) were purchased from Meixuan Biotechnology Co., Ltd. (Shanghai, China). MCF-7 and ZR-75-1 cells were grown and maintained in RPMI 1640 supplemented with 10% fetal bovine serum (Sigma) and 1% penicillin and streptomycin (Gibco, Carlsbad, NY, USA). MDA-MB-231, MDA-MB-231/PTX, BT549 and BT549/PTX cells were cultured in DMEM supplemented with 10% FBS in a humidified incubator at 37 °C with 5% CO_2_. Hesperadin (200 nM) and bisindolylmaleimide I (1 µM) purchased from Selleck (Selleck Chemicals, Shanghai, China) were used to treat cells in culture.

Cell transfection was carried out with Liposome 3000 reagent (Thermo Fisher Scientific, Inc., MA, USA). Transfections were performed in 24-well or 96-well plates with siRNAs (10 nM) or plasmids (10 ng/µL) according to the manufacturer’s suggestion. The wild-type expression plasmid pCMV-AURKB (NM_004217.2) was obtained from SinoBiological (Beijing, China). The plasmid pCMV-AURKB MUT, with an amino acid mutation at residue 227 (S to A), and the lentivirus-shRNA vector for AURKB were designed and synthesized by Hitrobio (Hitrobio.tech, Beijing, China). Lentiviral-mediated shRNA was performed using the shRNA lentiviral vector psiRNA-h7SK G1 (InvivoGen, San Diego, USA). The oligonucleotides containing the AURKB target sequence were inserted into the BbsI/BbsI cloning sites (forward, 5′-ACCTCGTGGGACACCCGACATCTTAATCAAGAGTTAAGATGTCGGGTGTCCCACTT-3′; reverse, 5′-CAAAAAGTGGGACACCCGACATCTTAACTCTTGATTAAGATGTCGGGTGTCCCACG-3′). PRKCE siRNA (5′-GGTGCCACGAGCTCATAATCA-3′) and NC siRNA (5′-GGAGTCTTTAGCACTAGAGGT-3′) were synthesized by GenePharma (GenePharma Co., Ltd., Shanghai, China).

### Cell viability assay

Cells were seeded into 96-well plates at a rate of 1 × 10^4^/well. After overnight incubation, the cells were subjected to different treatments and incubated for the specified period of time. At the end of the treatment, 100 µL of fresh medium containing 10% CCK-8 (Dojindo Laboratories, Kumamoto, Japan) was used to replace the medium in each well, and the culture was continued for an additional hour. The absorbance at 450 nm was measured with a microplate reader (PerkinElmer, USA). The experiment was repeated three times, and the average value of a single experiment was taken as the final experimental result.

### Quantitative real-time polymerase chain reaction (qRT-PCR)

Total RNA was extracted with TRIzol reagent (Invitrogen, MA, USA), and the concentration and purity of RNA were detected by UV spectrophotometry. cDNA was synthesized with a reverse transcription kit (Takara, Japan). The concentration of the reverse-transcribed cDNA product was diluted and adjusted to 50 ng/µL. Fluorescence-based quantitative PCR was carried out with GAPDH as the internal reference. The PCR conditions were as follows: 94 °C for 2 min; followed by 40 cycles (94 °C, 20 s; 60 °C, 35 s; 72 °C, 1 min) and a final step at 72 °C for 10 min. At the end of the reaction, the melting curve was analyzed to determine whether there was nonspecific amplification. The relative expression level of mRNA in cells was calculated by the quantitative 2^−ΔΔCt^ method. Three wells were set up for each group, and the average value was repeated 3 times in each group.

### Western blot assay

After treatment, RIPA lysis buffer (Sigma-Aldrich, St Louis, MO, USA) was added to the cells or exosomes of each group, the total protein was extracted, and the protein was quantified with a BCA kit (Biyuntian Biotechnology, Shanghai, China). After SDS-PAGE electrophoresis, the separated proteins were transferred to nitrocellulose (NC) membranes (Bio-RadRad, CA, USA). After blocking with 5% skim milk, the corresponding primary antibody was added, incubated overnight at 4 °C, and washed with TBST 3 times for 10 min each time. After adding horseradish peroxidase-coupled anti-rabbit or rat antibody (diluted 1:2000) and incubation at 37 °C for 1 h, the membrane was washed thoroughly 3 times, and each wash was performed for 10 min. Finally, protein bands were visualized using enhanced chemiluminescent substrate (Thermo Scientific). β-Actin was used as an internal reference, and the results were analyzed with ImageJ software. Primary antibodies against AURKB (ab45145, 1:1000), p-AURKB (ab210706), Lamin B1 (ab16048, 1:1000) and GAPDH (ab181602, 1:1000) were purchased from Abcam. Antibodies against HSP70 (sc-32239), CD81 (sc-166029), TSG101 (sc-7964), and calnexin (sc-46669) were purchased from Santa Cruz (Santa Cruz Biotechnology, Santa Cruz, CA, USA). The RAB27B antibody (#44813, 1:1000) was obtained from Cell Signaling Technology (Danvers, MA, USA). Antibodies against PRKCE (GTX133937, 1:500) and p-PRKCE (GTX105452, 1:500) were obtained from GeneTex (GeneTex Inc., CA, USA).

### Co-immunoprecipitation

The cells were lysed in 50 mM Tris–HCl (pH 8.0), 150 mM NaCl, 5 mM EGTA (pH 8.0), 50 mM NaF (pH 8.0), 10% glycerol, 1.5 mM MgCl_2_, and 1% Triton X-100, and the protein concentration was determined by the BCA method. One milligram of cell lysate was pretreated with protein A hand G magnetic beads (Millipore Corp., Bedford, MA, USA) and incubated with 5 µg anti-DDK antibody (AB205606, Abcam) or normal rabbit IgG (# 2729, Cell Signaling). The complex was incubated with protein Ahamg magnetic beads for 2 h. The immune complex was washed, eluted and denatured in Laemmli buffer. The immuno-precipitated proteins were transferred to nitrocellulose membranes after SDS gel electrophoresis. For immunoblotting, the primary antibody used was anti-RAB27B (# 44813, Cell Signaling).

### Exosomes extraction and identification

Conditioned medium was used throughout the cell culture process before exosome extraction. The conditioned medium was obtained as follows: fetal bovine serum (FBS) was centrifuged at 120,000*g* in a Beckman Coulter Optima XPN ultracentrifuge. (Beckman Coulter, Brea, CA, USA) with an SW 41 Ti rotor for 5 h to remove microbubble-like exosomes. The cells were harvested by centrifugation at 300*g* for 10 min, and the supernatant was collected. The acellular supernatant was then centrifuged at 2000*g* for 20 min, followed by 10,000*g* for 30 min to remove cells and fragments. The supernatant was filtered with a 0.22 μm membrane (Millipore, MA, USA) and centrifuged at 4 °C at 100,000*g* for 70 min in a Beckman ultracentrifuge to precipitate the exosomes. The precipitate was washed in a large volume of cold PBS and centrifuged again at 4 °C at 100,000*g* for 70 min. Finally, the exosomes were re-suspended in PBS for further analysis and functional studies.

The collected exosomes were re-suspended in 30–50 μL PBS. The purified solution of 10 μL exosomes was re-suspended and then dropped onto the carrier copper mesh and allowed to stand for 1 min. Then, 30 μL phosphotungstic acid solution (20 mL/L) was dropped onto the copper mesh and negatively stained at room temperature for 5 min. The copper mesh was baked under a self-burning lamp, and the images were observed under a transmission electron microscope (FEI Tecnai G2, Eindhoven, Netherlands).

### Quantitative analysis of exosomes

The prepared exosome samples were examined by nanoparticle tracking analysis (NTA). In brief, the exosomes re-suspended in 50 µL PBS were further diluted 300 times to reach 20 to 100 objects per frame and were detected with a NanoSight NS-300 (NanoSight Technology, Malvern, UK). Each sample was measured three times by the camera, the acquisition time was 30 s, and the detection threshold was set to 3. At least 200 completed projects were analyzed in each video. The results were captured and analyzed with NTA 2.3 software. The NTA measurements of exosome concentration (particles/mL) were normalized to the exosome-producing cell number.

### Quantitative analysis of paclitaxel in exosomes

Before isolation of the exosomes, the cells were incubated in conditioned medium without exosomes before and during the experiment. Paclitaxel (1 µM) was added to the cells for 2 h, and the cells were washed with PBS and then incubated in conditioned medium for 24 h. One-hundred microliters of exosome samples (dissolved in PBS) was spiked with a known concentration of internal standard and then extracted with 1 mL ethyl acetate. The ethyl acetate layer was separated and evaporated under reduced pressure. Then, the obtained samples were reassembled in 1 mL methanol and transferred to an HPLC vial. The sample was divided into equal 10 µL portions and injected into the HPLC–MS system. HPLC–MS was performed by Beijing Protein Innovation Co. Ltd. (Beijing, China).

### Protein stability assay

The stability of the protein was analyzed by the cycloheximide (CHX) chase method. CHX was purchased from MedChemExpress (Monmouth Junction, NJ, USA). CHX was dissolved in DMSO at 100 µg/mL and used in treated cell culture medium at 100 µg/mL for the indicated times. The level of RAB27B protein was detected by Western blotting. β-Actin was used as a reference.

### Mouse xenograft model

The breast cancer cell lines were labeled with luciferase by VIEWSOLID BIOTECH (Beijing, China). The labeled cells in logarithmic phase were digested with trypsin to prepare a single-cell suspension. The cells were washed with 1 × PBS three times, and the cell density was adjusted to 4 × 10^7^/mL. The tumor cell suspension was extracted with a 1 mL syringe and inoculated subcutaneously on one side of the back of six-week-old NOD-SCID mice. Each site was inoculated with 0.1 mL containing 4 × 10^6^ viable cells. Four weeks later, tumor growth was detected using an in vivo imaging system (PerkinElmer). After animal sacrifice, paraffin-embedded tissues from the xenotransplanted tumors were processed for H&E and IHC staining. All animal experiments were performed according to the Laboratory Animal Guidelines for Using Animals in the Education of the Chinese Association for Laboratory Animal Sciences (CALAS) and approved by the Animal Use and Care Committee of Beijing Hospital of Traditional Chinese Medicine Affiliated to Capital Medical University (No. 20200057).

### Statistical analysis

All statistical analyses were performed in GraphPad Prism 8.0 (GraphPad Prism Software, San Diego, CA, USA). Data are expressed as the mean ± SD. A two-tailed Student's *t* test was used to compare two groups of normally distributed data. For multi-group comparisons, normally distributed data were analyzed using 1- or 2-way ANOVA. Statistical significance was defined as a *P* value ≤ 0.05.

## Results

### Abnormally high expression of AURKB in breast cancer tissues and cells

**Fig. 1:**
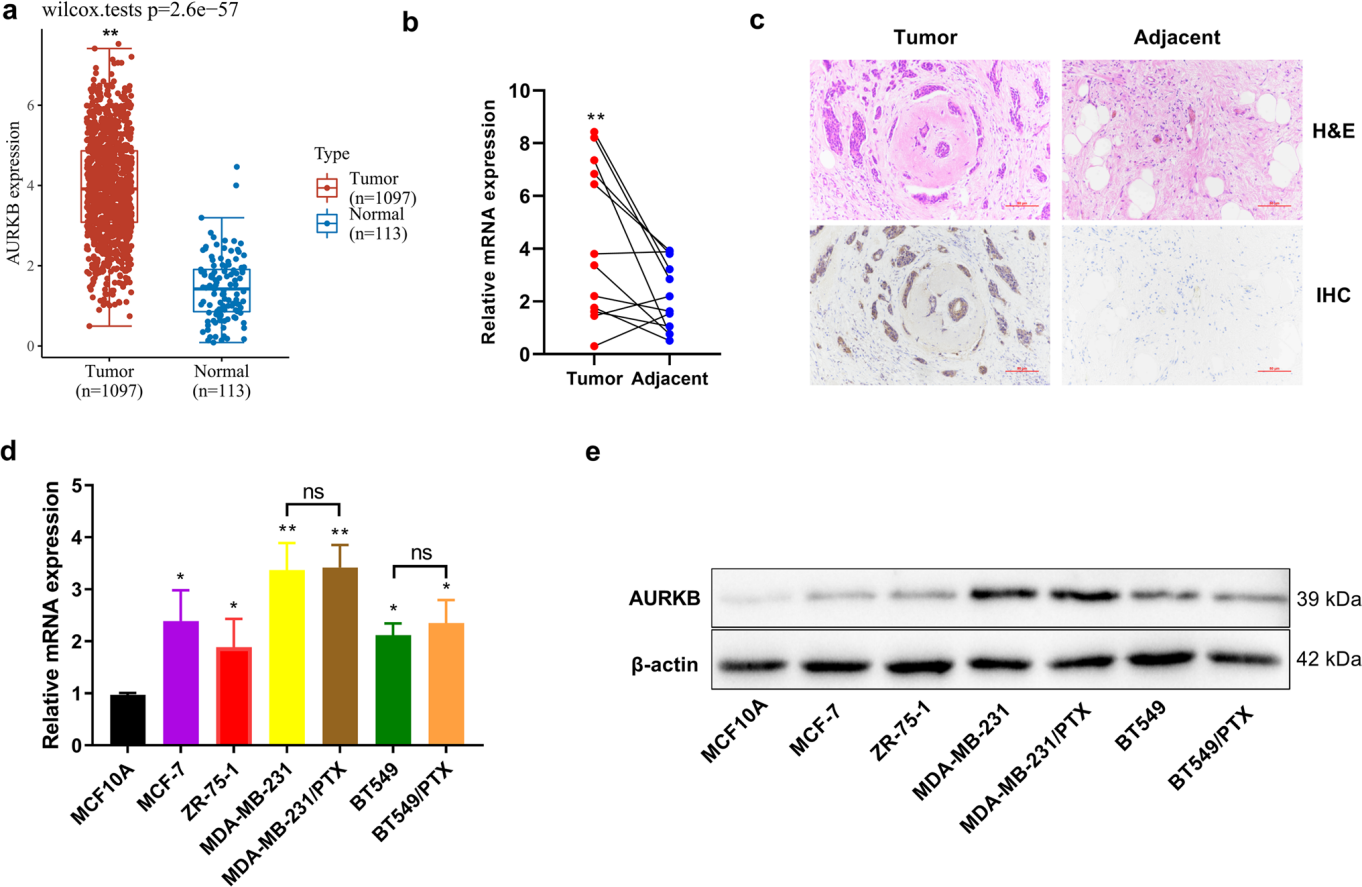
Analysis on the expression of AURKB in breast cancer tissues and cells. **a** Analysis of AURKB mRNA expression difference in breast cancer tissues and normal control tissues by online tool ACLBI. **b** Analysis of the relative content of AURKB in 15 pairs of breast cancer tissues and corresponding para-cancerous tissues using quantitative PCR. **c** Detection of the expression of AURKB in breast cancer tissues and adjacent tissues using H&E and IHC staining. Scale bar, 50 μm. **d**, **e** Detection of the relative expression levels of AURKB mRNA and protein in multiple breast cancer cells and normal breast epithelial cells by quantitative PCR and Western blot. Paired *t* test and one-way ANOVA with Tukey post hoc analysis. *P < 0.05, **P < 0.01, and *ns* no significant difference

According to the analysis of breast cancer data in TCGA using the online tool ACLBI (https://www.aclbi.com/), AURKB was abnormally highly expressed in breast cancer tissues (Fig. 1a). Then, detection of the collected clinical specimens by quantitative PCR showed that the relative content of AURKB mRNA in breast cancer tissues was significantly higher than that in the corresponding adjacent tissues (Fig. 1b). Similarly, the AURKB protein was relatively highly expressed in breast cancer tissues, as revealed by immuno-histochemical detection (Fig. 1c). Subsequent analysis focused on the expression of AURKB in breast cancer cells. Both quantitative PCR and Western blot analysis indicated that the relative AURKB mRNA and protein levels were significantly higher in non-triple -negative breast cancer cells (MCF-7, ZR-75-1), triple-negative breast cancer cells (MDA-MB-231 and BT549), and PTX-resistant MDA-MB-231/PTX and BT549/PTX cells than in immortalized normal breast epithelial cells (Fig. 1d, e).

### Effect of the phosphorylation level of AURKB on PTX resistance of breast cancer cells

**Fig. 2:**
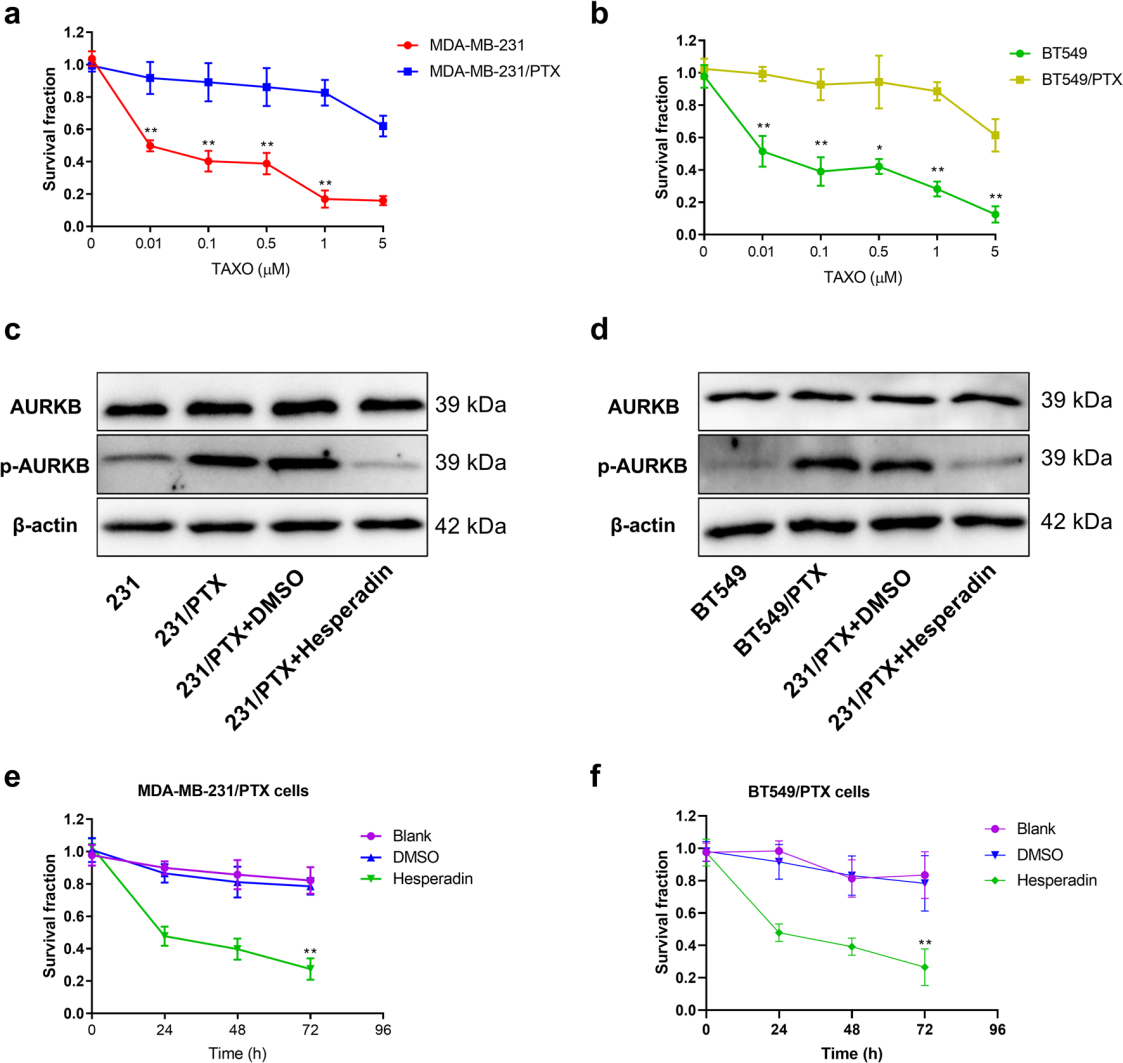
Correlation between AURKB expression level and resistance to PTX in breast cancer. **a**, **b** Measurement of cell viability by CCK-8 in breast cancer cells (MDA-MB-231 and BT549) and corresponding PTX-resistant cells (MDA-MB-231/PTX and BT549/PTX) that were treated with PTX at different concentrations for 48 h. **c** The relative contents of AURKB and phosphorylated AURKB in PTX-resistant cell line MDA-MB-231/PTX and wild-type cell line MDA-MB-231 as well as after Hesperadin (50 μM) treatment by Western blot. **d** The relative contents of AURKB and phosphorylated AURKB in PTX-resistant cell line BT549/PTX and wild-type cell line BT549 as well as after Hesperadin treatment by Western blot. **e**, **f** The cell survival rates of blank group, DMSO group and Hesperadin (200 nm) treatment group by CCK-8 at different time points after MDA-MB-231/PTX and BT549/PTX were treated by PTX (0.5 μM). Comparisons among groups were determined by one-way ANOVA with Tukey post hoc analysis. *P < 0.05 and **P < 0.01

Further analysis was carried out to investigate the difference in resistance between breast cancer cells (MDA-MB-231 and BT549) and their resistant cell lines (MDA-MB-231/PTX and BT549/PTX) to PTX (Fig. 2a, b). However, there was no significant difference in the relative expression of AURKB mRNA and protein between the two groups of PTX-resistant and wild-type cells (Fig. 1d, e). We subsequently detected was the phosphorylation level of AURKB in different cells. Although there was no significant difference in the total protein content of AURKB between PTX-resistant and wild-type cell lines, the level of phosphorylated AURKB (phospho S227) in PTX-resistant cells was significantly higher than that in wild-type cells (Fig. 2c, d). These results suggested that the phosphorylation of AURKB might be related to PTX resistance. Then, an AURKB inhibitor (hesperadin) was used in PTX-resistant cells. Consequently, hesperidin simultaneously inhibited the phosphorylation of AURKB and reduced drug resistance (Fig. 2e, f).

**Fig. 3:**
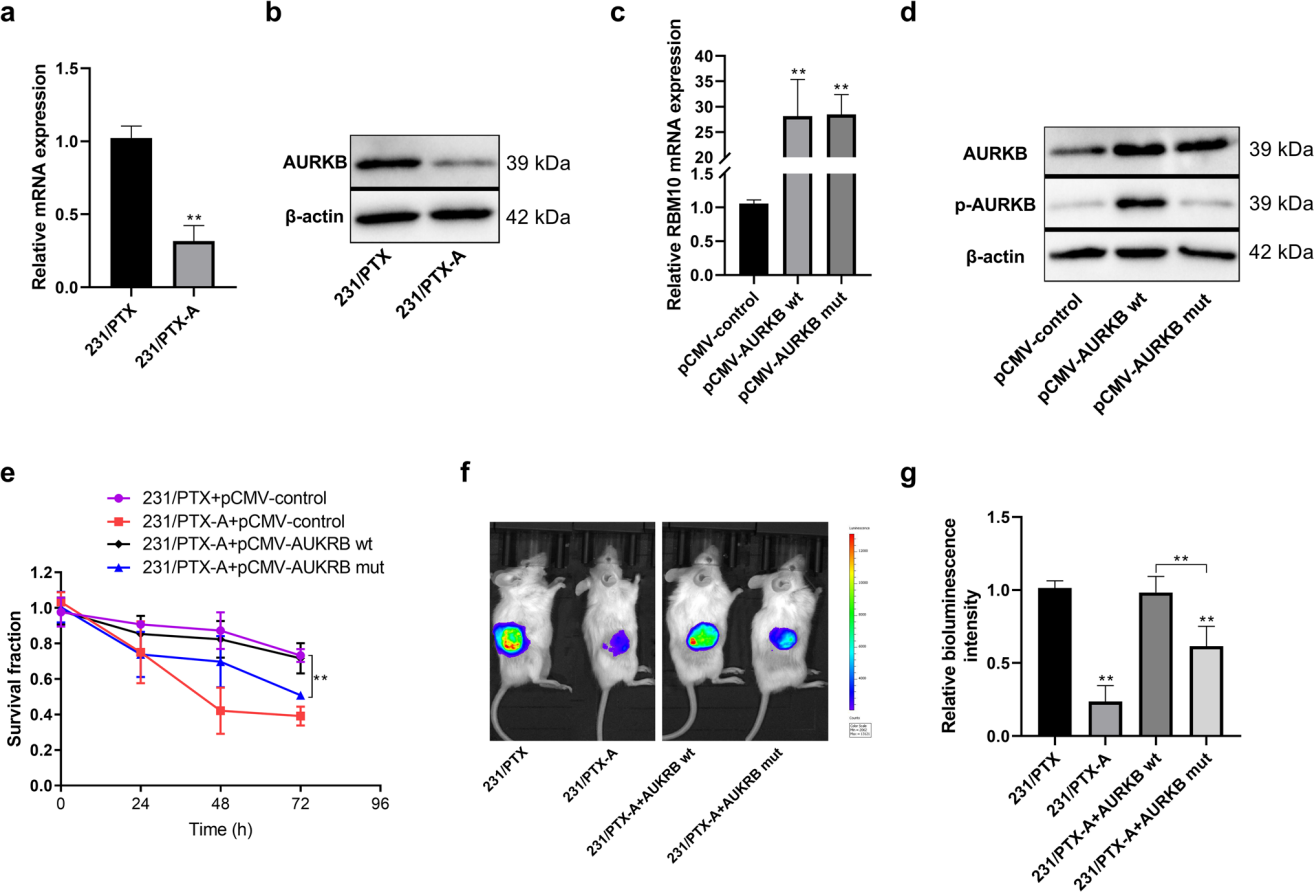
Effect of the phosphorylation level of AURKB on PTX resistance of breast cancer cells. **a**, **b** The relative expression levels of AURKB mRNA and protein in MDA-MB-231/PTX-resistant and AURKB stable knockdown cells (231/PTX-A) by quantitative PCR and Western blot. **c**, **d** The relative expression levels of AURKB mRNA and protein in AURKB stable knockdown cells (231/PTX-A) transfected with wild-type and mutant-type expression plasmids for 48 h by quantitative PCR and Western blot. **e** Detection of the survival rate in different groups at different time points using CCK-8 method after cell treatment with PTX (0.5 μM); **f**, **g** The cells labeled with luciferase were xenografted subcutaneously into NOD-SCID mice. At the fourth week, the fluorescence intensity of subcutaneous tumor was examined by in vivo imaging. One-way ANOVA with Tukey post hoc analysis. *P < 0.05 and **P < 0.01

To verify the correlation between the AURKB phosphorylation level and resistance to PTX in breast cancer cells, a lentivirus-shRNA vector was used to prepare an MDA-MB-231/PTX-resistant cell line with stable knockdown of AURKB (Fig. 3a, b), i.e., 231/PTX-A. When wild-type AURKB was overexpressed in cells with stable knockdown of AURKB (Fig. 3c, d), the downregulated drug resistance of this cell line was significantly restored (Fig. 3e), yet no such effect was observed after overexpression of mutant-type AURKB (S227 → L) (Fig. 3e). Furthermore, four cell lines were labeled with firefly luciferase, including the MDA-MB-231/PTX-resistant cell line, stable AURKB knockdown cell line (231/PTX-A) and wild-type and mutant AURKB overexpression cell lines. NOD/SCID mice were used to construct a xenogeneic tumor transplantation model to study drug resistance to PTX (Fig S1). The results revealed that the tumor volume of the stable AURKB knockdown group was significantly smaller than that of the PTX-resistant group, and the tumor volume was also significantly lower in the mutant-type AURKB overexpression group than in the wild-type overexpression group (Fig. 3f, g). Collectively, these results suggest that the phosphorylation level of AURKB in breast cancer cells has a strong association with resistance to PTX.

### Regulatory role of PRKCE in the phosphorylation level of AURKB in PTX-resistant breast cancer cell line

**Fig. 4:**
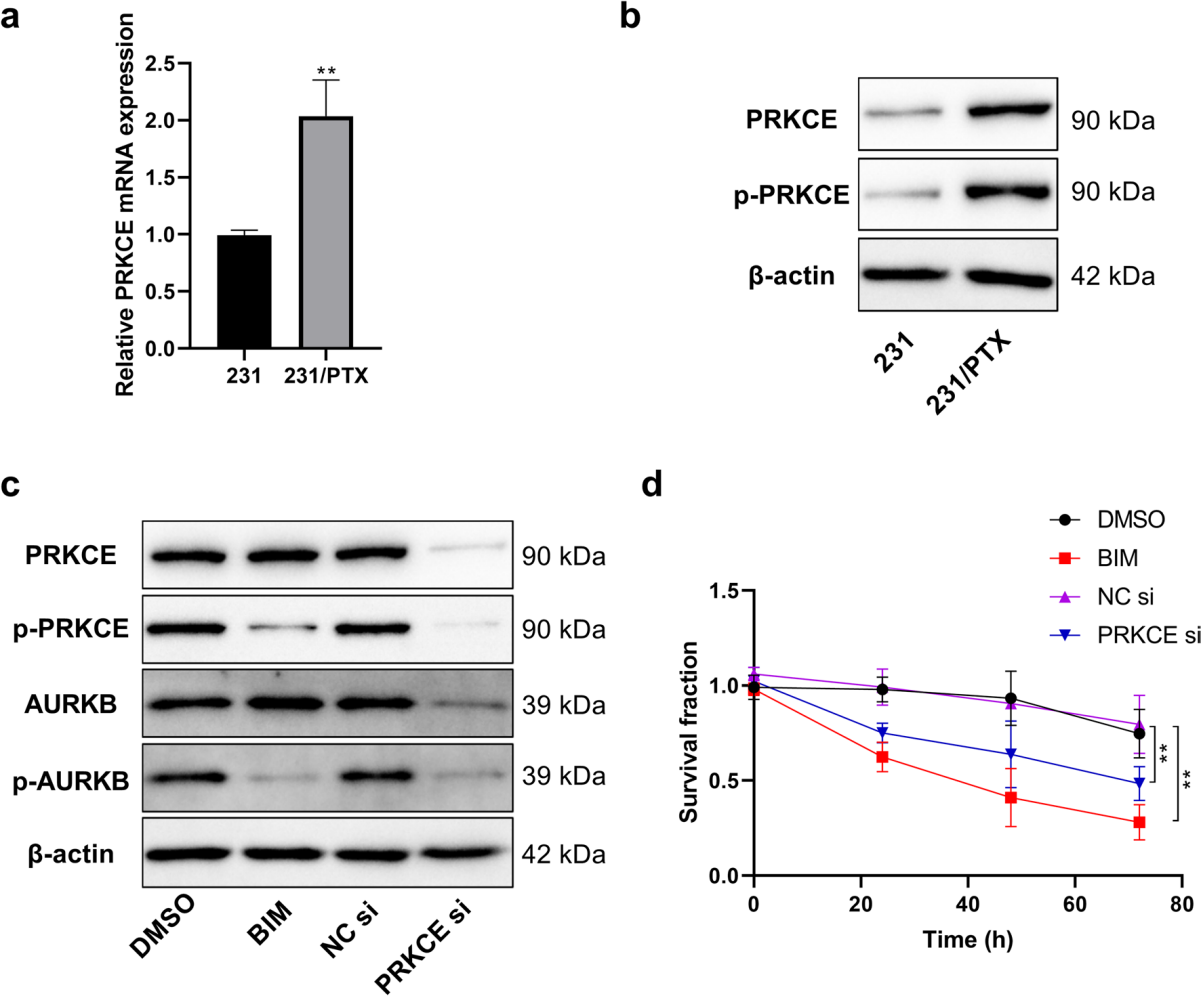
Regulatory role of PRKCE in the phosphorylation level of AURKB. **a**, **b** Analysis of PRKCE mRNA and protein expression differences in breast cancer cell MDA-MB-231 and corresponding PTX-resistant MDA-MB-231/PTX using quantitative PCR and Western blot. **c** Relative contents of PRKCE and phosphorylated PRKCE using Western blot after MDA-MB-231/PTX cells were treated with Bisindolylmaleimide I (1 μM) or PRKCE siRNA (20 nM) for 24 h. **d** Detection of the survival rate in different groups at different time points using CCK-8 method after cell treatment with PTX (0.5 μM) when MDA-MB-231/PTX cells were treated with Bisindolylmaleimide I (1 μM) or PRKCE siRNA (20 nM) for 24 h. One-way ANOVA with Tukey post hoc analysis. *P < 0.05 and **P < 0.01

Pike et al. reported that the phosphorylation of AURKB s227 in human colon cancer cells (DLD1) and human embryonic kidney cells (293) was regulated by phosphokinase PRKCE [19]. In our study, related experiments were performed to compare the relative expression of PRKCE mRNA and protein in MDA-MB-231 breast cancer cells and the corresponding PTX-resistant MDA-MB-231/PTX cells. PRKCE mRNA and protein expression levels, as well as protein phosphorylation levels, were higher in PTX-resistant cells than in wild-type cells (Fig. 4a, b). Subsequently, PTX-resistant MDA-MB-231/PTX cells were treated with PRKCE siRNA or PRKCE inhibitor (bisindolylmaleimide I, BIM). Both treatments effectively inhibited the phosphorylation of AURKB (Fig. 4c) and reduced the drug resistance of cells (Fig. 4d).

### Effect of phosphorylation on the cellular localization of AURKB

**Fig. 5:**
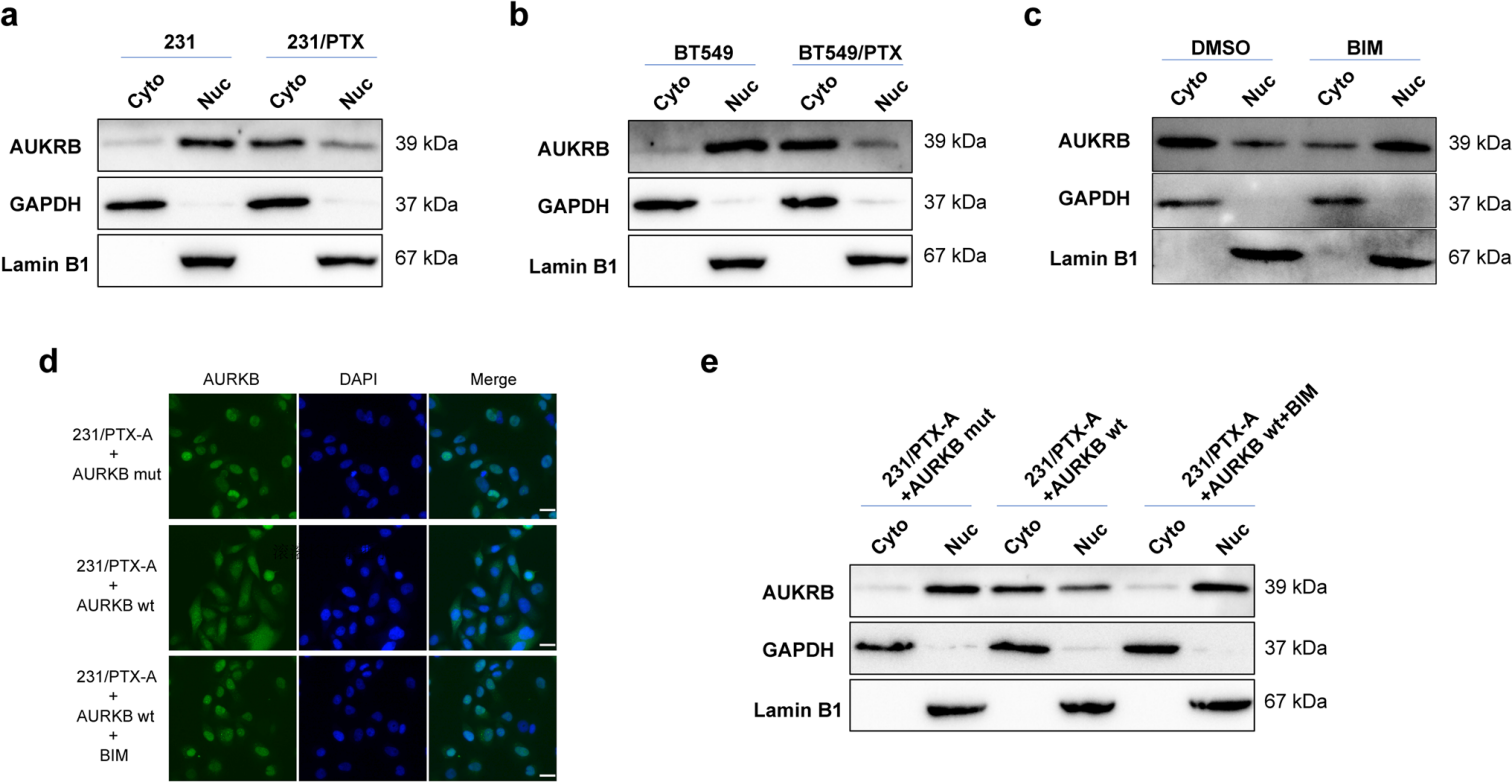
Effect of phosphorylation on the cellular localization of AURKB. **a** Analysis of the distribution differences of AURKB in the cytoplasm (normalized to GAPDH) and nucleus (normalized to Lamin B1) of 231 and 231/PTX cells using Western blot after the extraction of cytoplasm and nucleus, respectively. **b** Analysis of the distribution differences of AURKB in the cytoplasm and nucleus of BT549 and BT549/PTX cells using Western blot. **c** Analysis of the distribution differences of AURKB in the cytoplasm and nucleus after 48 h of DMSO and bisindolylmaleimide I (1 μM) treatment of 231/PTX cells using Western blot analysis. **d** Analysis of the subcellular localization of wild-type and mutant-type AURKB using immunofluorescence after the transfection of overexpression plasmid of AURKB in 231/PTX-A cells with stable knockdown of AURKB for 48 h. Scale bar, 20 μm. **e** The distribution difference of wild-type and mutant-type AURKB in cytoplasm and nucleus using Western blot after the transfection of overexpression plasmid of AURKB in 231/PTX-A cells for 48 h

It has been recognized that the phosphorylation level of AURKB is associated with resistance to PTX in breast cancer cells. However, we know nothing about the specific mechanism by which AURKB phosphorylation influences the drug resistance of breast cancer cells. In our subsequent experiment, it was observed that there was a significant difference in the subcellular localization of AURKB in wild-type breast cancer cells (MDA-MB-231 and BT549) and the corresponding PTX-resistant cells. In wild-type cells, AURKB tended to be distributed in the nucleus (Fig. 5a, b), while in drug-resistant cells, AURKB was widely distributed in both the cytoplasm and nucleus (Fig. 5a, b). Next, we found that in PTX-resistant cells, the PRKCE inhibitor BIM could alter the cellular localization of AURKB, making it more likely to be distributed in the nucleus (Fig. 5c). In other words, BIM may alter the cellular localization of AURKB by inhibiting its phosphorylation. Furthermore, in 231/PTX-A cells with stable knockdown of AURKB, the distribution of wild-type AURKB in the cytoplasm was much higher than that of mutant AURKB after overexpression of wild-type and mutant AURKB (Fig. 5d, e). Simultaneously, the use of BIM could effectively restrict the cytoplasmic distribution of wild-type AURKB (Fig. 5d, e). These results suggest that the phosphorylation of AURKB may affect the cellular localization of AURKB. Wild-type AURKB is phosphorylated via the action of PRKCE, and phosphorylated AURKB is able to undergo a change in distribution from the nucleus to the cytoplasm, while AURKB with mutations in the phosphorylation site stays in the nucleus.

### Involvement of AURKB in the regulation of RAB27B expression

**Fig. 6:**
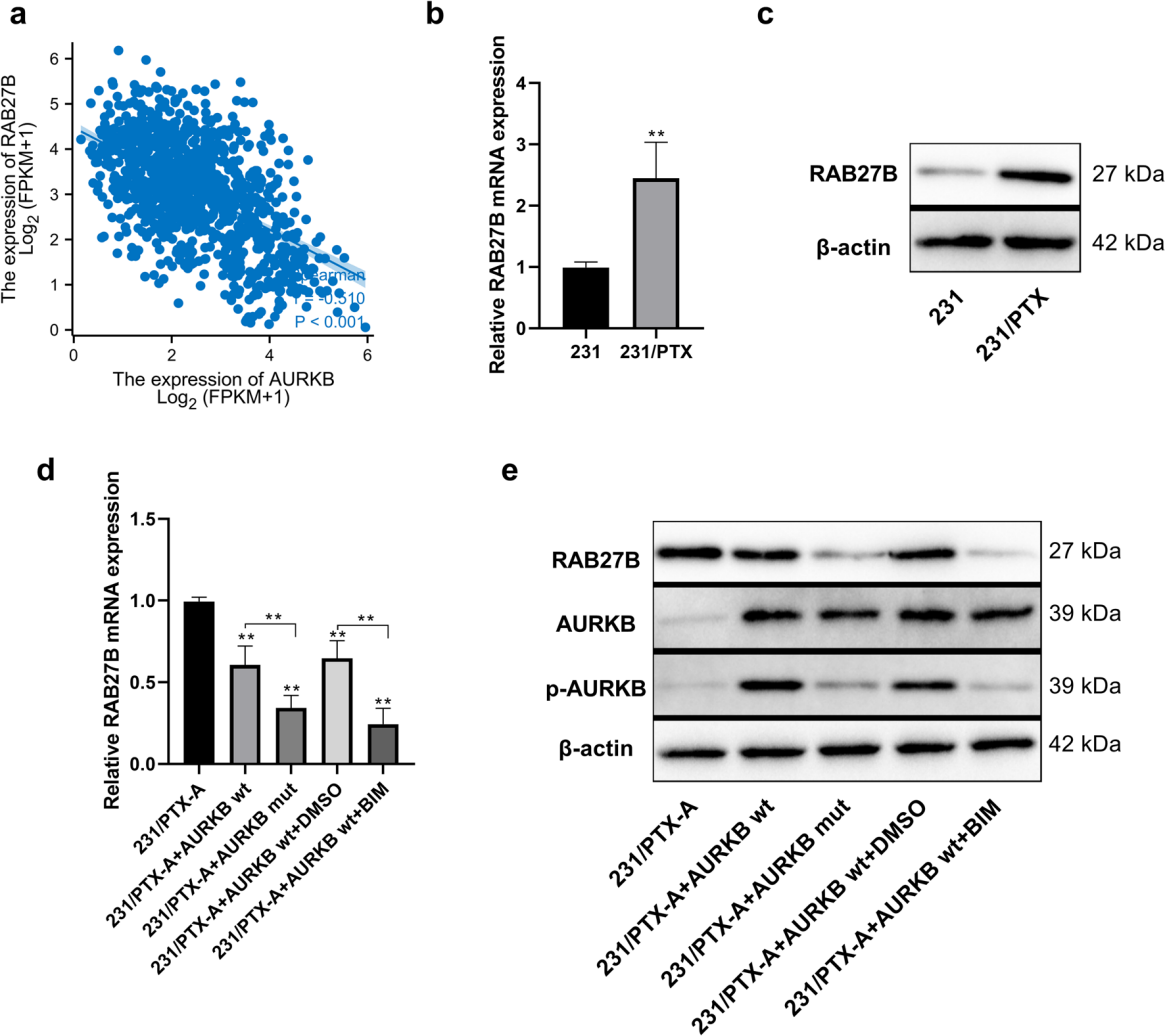
Correlation analysis of AURKB and RAB27B expression. **a** Correlation analysis between AURKB mRNA and RAB27B mRNA expression using online tool UALCAN to collect breast cancer data in TCGA. **b**, **c** Analysis of RAB27B mRNA and protein expression differences in breast cancer cells MDA-MB-231 and corresponding PTX-resistant MDA-MB-231/PTX cells using quantitative PCR and Western blot. **d**, **e** Analysis of the mRNA and protein expression of RAB27B using PCR and Western blot after the transfection with wild-type or mutant-type AURKB expression plasmids in 231/PTX-A cells with stable AURKB knockdown, and simultaneous treatment with DMSO or Bisindolylmaleimide I (1 μM) for 24 h. One-way ANOVA with Tukey post hoc analysis. *P < 0.05 and **P < 0.01

After using the online tool UALCAN (http://ualcan.path.uab.edu/) to collect breast cancer data in TCGA, the corresponding analysis revealed that AURKB mRNA was negatively correlated with RAB27B mRNA expression in breast cancer tissues (Fig. 6a, Table S1). Afterward, although there was no significant difference in AURKB mRNA and protein expression between MDA-MB-231 breast cancer cells and their corresponding PTX-resistant MDA-MB-231/PTX cells (Fig. 1d, e), there was a significant difference in the expression level of RAB27B (Fig. 6b, c). In addition, the relative content of RAB27B in the drug-resistant cell line was higher than that in the wild-type cell line at both the mRNA and protein levels. Conversely, wild-type or phosphorylated mutant AURKB was overexpressed in the 231/PTX-A cell line with stable knockdown of AURKB. The results showed that overexpression of both wild-type and mutant AURKB decreased the expression of RAB27B, while the inhibitory effect of wild-type AURKB on RAB27B expression was significantly weaker than that of mutant AURKB (Fig. 6d, e). In addition, the use of BIM, an inhibitor of PRKCE, enhanced the inhibitory effect of wild-type AURKB (Fig. 6d, e). These results confirm the negative regulation of RAB27B expression by AURKB and suggest that phosphorylation of AURKB can weaken this proposed negative regulatory role.

**Fig. 7:**
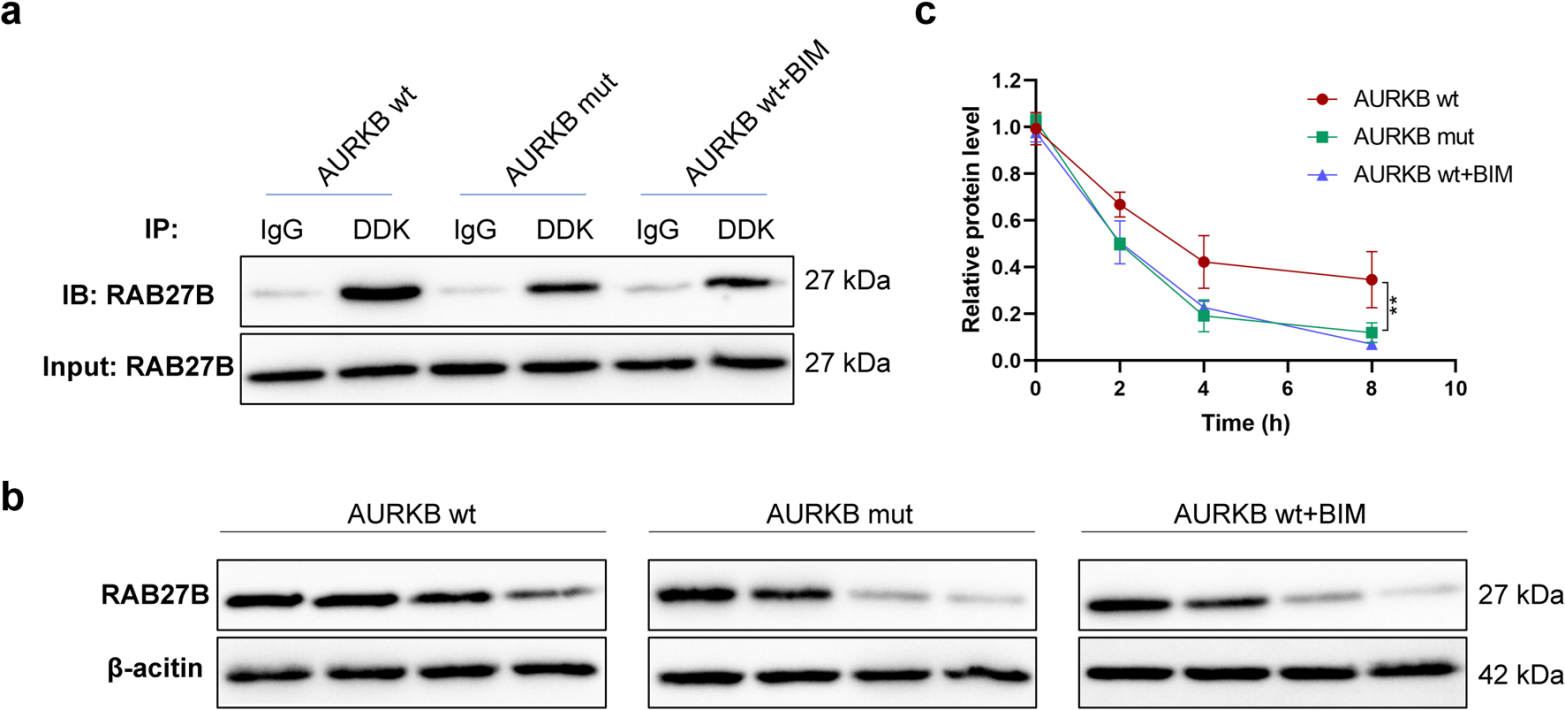
Interaction between AURKB and RAB27B. **a** Detection of the interaction between AURKB and RAB27B by immunoprecipitation assay using DDK-labeled antibody after the transfection with wild-type or mutant-type AURKB expression plasmids in 231/PTX-A cells with stable AURKB knockdown. **b** Detection of the relative content of RAB27B protein at different time points after the transfection of 231/PTX-A with wild-type or mutant-type AURKB expression plasmid for 24 h, and subsequent treatment with Cycloheximide (100 μg/mL). **c** Quantification of RAB27B protein levels (normalized to β-actin) at different time points. One-way ANOVA with Tukey post hoc analysis. *P < 0.05 and **P < 0.01

Furthermore, there was also a potential interaction between AURKB and RAB27B at the protein level (Fig. S2, Table S2) according to data analysis based on the Protein–Protein Interaction Database STRING (https://string-db.org/) and BioGRID (https://thebiogrid.org/). After overexpression of wild-type or phosphorylated mutant AURKB in 231/PTX-A cells, immunoprecipitation was performed with a DDK-labeled antibody. Consequently, the relative amount of RAB27B co-precipitated with wild-type AURKB was significantly higher than that co-precipitated with mutant AURKB (Fig. 7a), and the use of the PRKCE inhibitor BIM reduced the co-precipitation advantage of wild-type AURKB (Fig. 7a). More interestingly, after overexpression of wild-type or phosphorylated mutant-type AURKB in 231/PTX-A cells, the stability of the RAB27B protein in wild-type AURKB overexpression group was significantly higher than that in mutant-type AURKB group after the addition of cycloheximide to inhibit protein translation (Fig. 7b, c). The above results suggest that AURKB regulates the expression of AURKB at the posttranscriptional level, and there was an intimate association of AURKB phosphorylation with the stability of the RAB27B protein. Overall, AURKB exerts a negative regulatory effect on RAB27B at the transcriptional level, while AURKB can weaken the negative regulation of RAB27B transcription by reducing its nuclear localization through phosphorylation, and phosphorylated AURKB can enhance the stability of RAB27B. In other words, RAB27B can attenuate the negative regulation of RAB27B via phosphorylation, which seems to be a compensatory mechanism.

### AURKB participates in exosome-mediated PTX resistance

**Fig. 8:**
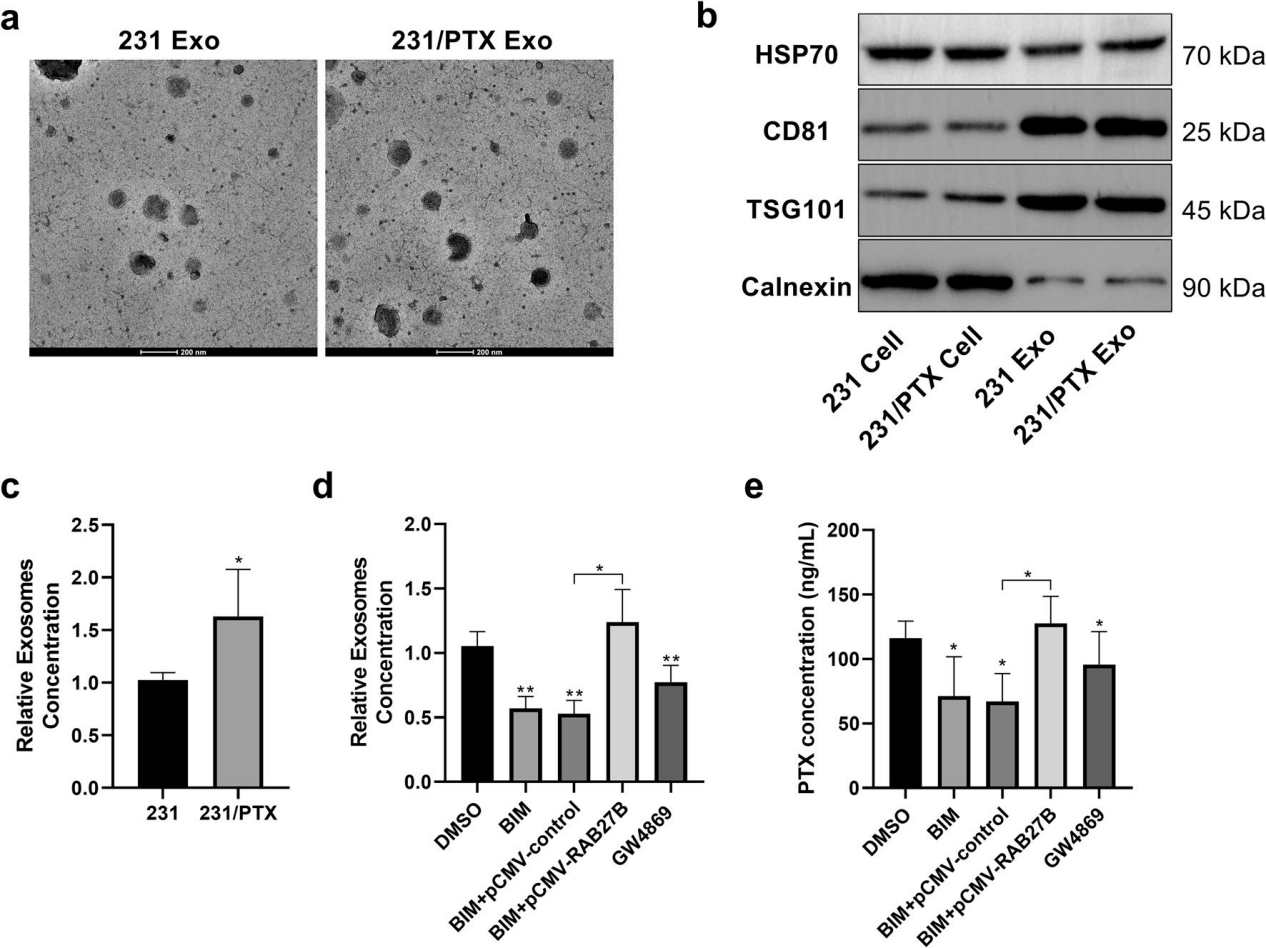
Involvement of AURKB in the regulation of exocrine secretion and PTX efflux in breast cancer cells. **a** Transmission electron micrographs of exosomes derived from MDA-MB-231 and MDA-MB-231/PTX cells. **b** Detection of related marker proteins in exosomes using Western blot. **c** Histogram of the relative exosome concentrations of MDA-MB-231 and MDA-MB-231/PTX cells after measurement of the relative exosome concentrations of MDA-MB-231 and MDA-MB-231/PTX cells by nanoparticle tracking analysis (NTA) and normalization with the number of cells. **d** Detection of the relative exocrine concentration in MDA-MB-231/PTX cells treated with BIM and transfected with RAB27B expression plasmid for 24 h; the sphingomyelinase inhibitor GW4869 was used as a positive control of inhibiting exosome secretion. **e** Measurement of the content of PTX in exosomes by HPLC–MS after the extraction of exosomes from the same culture supernatant when MDA-MB-231/PTX were treated with BIM and transfected with RAB27B expression plasmid for 24 h, and then treated by PTX (0.5 μM) for 2 h

**Fig. 9:**
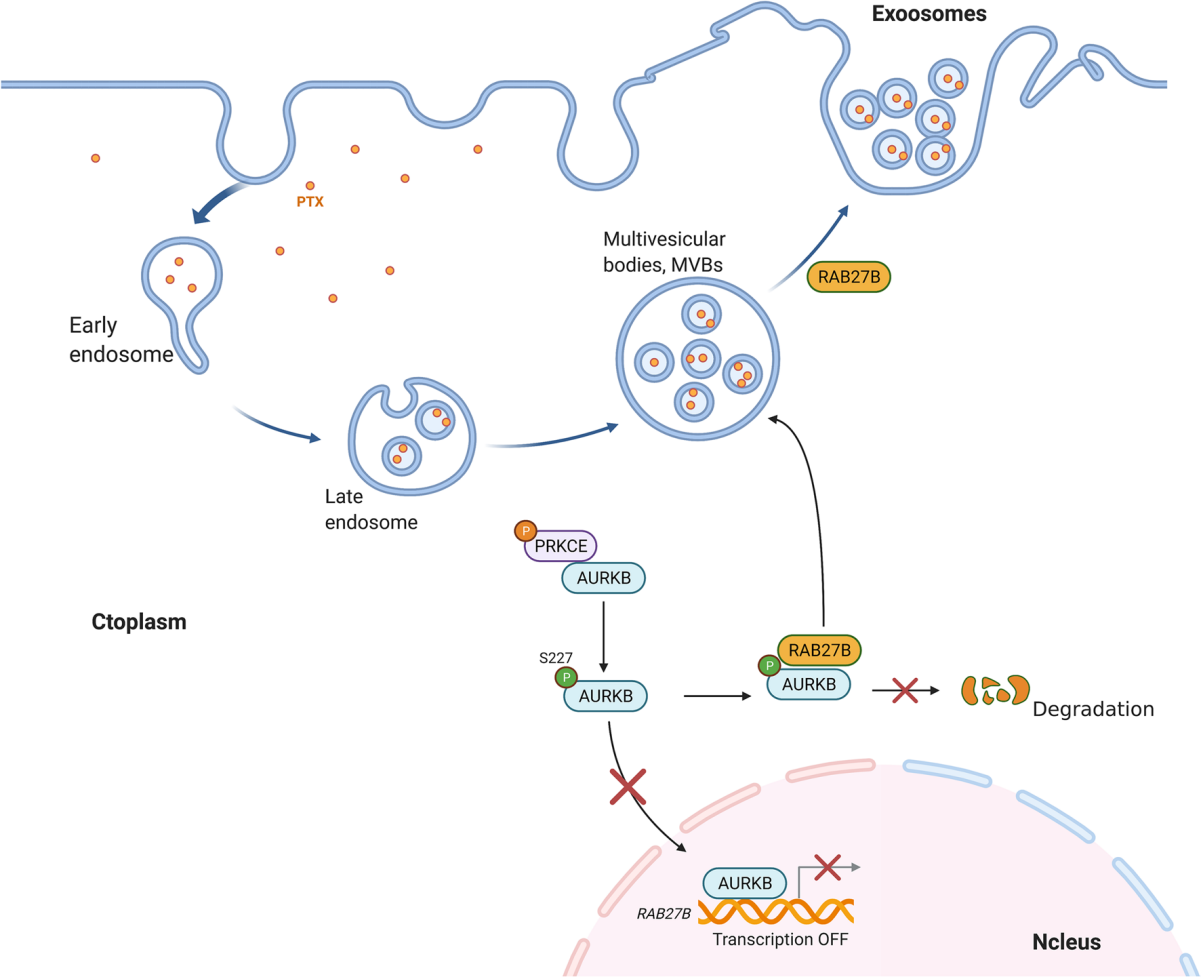
The pattern diagram to reveal that AURKB is involved in exosome release and PTX efflux through the regulation of RAB27B expression. (Created with BioRender.com)

RAB27B belongs to the Rab protein family, the largest subfamily of the Ras protein superfamily, which is an important participant in the process by which membrane-bound proteins are involved in vesicle transport and fusion [20]. It has been documented that RAB27B has an association with the outward transport of multi-vesicular bodies (MVBs) from the cytoplasm to the cell membrane, which mainly affects the secretion regulation of exosomes [21]. A growing number of studies have confirmed that exosomes are closely related to drug resistance. In our experiment, exosomes were purified and extracted from the wild-type MDA-MB-231 cell line and the drug-resistant MDA-MB-231/PTX cell line (Fig. 8a, b), and the relative exosome concentrations (exosome concentration/cell count) of the two cell lines were compared simultaneously. The exosome concentration of the drug-resistant cell line was significantly higher than that of the wild-type cell line (Fig. 8c). In the drug-resistant MDA-MB-231/PTX cell line, BIM, an inhibitor of PRKCE, effectively inhibited the secretion of exosomes (Fig. 8d), while simultaneous overexpression of RAB27B counteracted the inhibitory effect of BIM (Fig. 8d). These results suggest that BIM reduces the phosphorylation of AURKB by inhibiting the activity of PRKCE, and the absence of phosphorylation allows AURKB to stay in the nucleus and negatively regulate RAB27B at the transcriptional level, thus reducing the expression of RAB27B to inhibit the secretion of cellular exosomes. Furthermore, after drug-resistant MDA-MB-231/PTX cells were treated with PTX, the content of PTX in the exosomes in the same volume of culture medium was determined by HPLC–MS. Similar to the concentration of exosomes, the concentration of PTX in the BIM-treated group was significantly lower than that in the control group, while overexpression of RAB27B could restore the inhibitory effect of BIM (Fig. 8e). The above results suggest that AURKB is involved in exosome release and PTX efflux by regulating RAB27B expression (Fig. 9).

## Discussion

Great advancements have been made in breast cancer treatment technologies in the past thirty years. However, breast cancer is still a fatal cancer in female patients [22]. Chemotherapy is still the major choice for the treatment of these fatal diseases, especially for patients in the advanced stage [23, 24]. However, the emergence of drug resistance can be a major obstacle to improve the effectiveness of cancer treatment to a great extent. Even more troublesome cancer cells that are resistant to a single chemotherapeutic agent may frequently develop resistance to multiple structural, functional and mechanism-independent anticancer agents. It is known as multiple drug resistance (MDR), which is induced relatively complicated and multifactorial mechanisms. MDR is mainly divided into two categories of the classic and non-classical drug transport mechanisms. The classic mechanism of MDR is related to the involvement of P-glycoprotein (P-gp), which has been extensively explored [25]. There are many mechanisms of non-classical MDR, such as glutathione S-transferase (GST) and protein kinase C (PKC), blockade of the apoptosis pathway, topoisomerase and enhanced DNA repair ability [26]. Great progress has been made in research on tumor resistance. Nevertheless, in view of the highly complicated pathogenesis of malignant tumors, there is still a long way to go to achieve a real cure, and the regulation of P-gp alone cannot solve this problem. With respect to the above, while searching for P-gp reversal agents with high efficiency and low toxicity, efforts should also be made to find other drug resistance targets and their reversal agents.

PKC is a Ca^2+^-dependent protein kinase, and it is an important factor for intracellular signal transduction. It plays an essential role in mediating cell growth, differentiation and death [27]. At present, there are at least 13 subtypes of PKC after isolation and purification, among which subtypes α and β have aroused widespread concern. It has been documented that PKC can activate the phosphorylation of P-gp, enhance the function of P-gp, and then reduce drug accumulation [28]. The use of PKC inhibitors can partially reverse the MDR phenotype. For instance, Masanek et al. reversed drug resistance by inhibiting PKCα and PKCβ in their study, and the degree of reversal was similar to that achieved by inhibiting P-gp, indicating the existence of a relationship between PKC and MDR [29]. In addition, the activity of PKCα in multidrug-resistant MCF-7/ADR breast cancer cells was higher than that in MCF-7 cells (sensitive strain). The accumulation of doxorubicin was increased when the drug-resistant strain was treated with Ro-31-7549, an inhibitor of PKCα, suggesting that the activity of PKCα was positively correlated with drug resistance [30].

Protein kinase C-epsilon (PRKCE) is a member of the PKC family. Recent studies have revealed that PRKCE is an anti-apoptotic gene that is commonly upregulated to promote the survival of different types of cancer [31–34]. Moreover, according to existing research, downregulation of the PRKCE gene can decrease the proliferation potential and tumor formation ability of renal cell carcinoma in vivo [35]. In addition, PRKCE can also enhance drug resistance by phosphorylating extracellular signal regulated kinase (ERK), signal transducer and activator of transcription 3 (STAT3), activating transcription factor 2 (ATF2), phosphatidylinositol-3 kinase (PI3K) and P-gp [36–38]. In our experiment, the expression of PRKCE in PTX-resistant breast cancer cells was significantly higher than that in wild-type cells. Simultaneously, the findings obtained in our study suggest that the overexpression of PRKCE may be a potential cause of the increased phosphorylation of AURKB in drug-resistant cells (Fig. 4).

Furthermore, AURKB kinase has been confirmed to have a close relationship with the formation of malignant tumors. Its high expression may induce centrosomal amplification, mitosis failure, polyploid formation and loss of the p53 gene. A substantial number of studies have reported that AURKB is a tumor-related gene. To date, it has been found that AURKB is highly expressed in various malignant tumors, including colon adenocarcinoma, thyroid follicular carcinoma, laryngeal carcinoma, lung cancer, and breast invasive ductal carcinoma [39–42]. In our experiment, a significant difference in the phosphorylation level of AURKB was found between PTX-resistant breast cancer cells and wild-type cells, rather than the total expression of AURKB. The findings of our study confirm that there is a positive association between the phosphorylation of AURKB and resistance to PTX, both in vivo and in vitro. Our results also demonstrate that in PTX-resistant breast cancer cell lines, either direct inhibition or indirect reduction of AURKB phosphorylation levels [including shRNA knockdown of AURKB expression, direct inhibition of AURKB activity using hesperadin, mutation of AURKB phosphorylation site (Fig. 3e), or use of PRKCE siRNA or PRKCE inhibitor BIM to indirectly inhibit AURKB phosphorylation] are effective in reversing cellular drug resistance.

In recent decades, great concern has been aroused by the involvement of exosomes in the occurrence of tumor drug resistance. Exosomes are extracellular vesicles (EVs) that are secreted by various cells of the organism and participate in physiological and pathological activities. Exosomes can mediate specific cell-to-cell interactions and activate related signaling pathways to deliver functional molecules, such as microRNAs (miRNAs) and proteins [43]. For example, it has been reported that high expression of miR-221 in breast cancer may induce resistance to trastuzumab [44], while the upregulation of miR-21 and downregulation of miR-133b can reduce the sensitivity of lung cancer cells to cisplatin [45]. Furthermore, P-gp transfer mediated by exosomes has been found to be a major mechanism contributing to docetaxel resistance in breast cancer cells [46, 47]. In view of the aforementioned findings, functional molecules, such as miRNAs and proteins, carried by exosomes may be associated with tumor drug resistance. This suggests that exosomes can be considered signal transduction mediators involved in the sensitivity of tumors to chemotherapeutics. In addition, exosomes can also directly transport chemotherapeutics out of tumor cells, leading to drug resistance [48]. In our study, in PTX-resistant breast cancer cells, AURKB weakened the negative transcriptional regulation of RAB27B by self-phosphorylation, while phosphorylation of AURKB further enhanced the stability of RAB27B. Furthermore, owing to the increase in the relative content of RAB27B, there was an increase in both the number of exosomes released from drug-resistant cells and the concentration of exosomes released in response to PTX. In other words, AURKB interacts with the exosome secretion pathway in a phosphorylation-dependent manner to mediate drug resistance. However, our study emphasized the relationship between exosome concentration and drug resistance. It is still unknown whether AURKB phosphorylation affects the changes in other effector molecules in exosomes, which also deserves further exploration.

The Rab protein family is the largest subfamily of the Ras superfamily and is an important participant in vesicle transport and fusion. The Rab protein has alternative forms: the active GTP-binding and inactive GDP-binding states. Ostrowski et al. identified 5 Rab GTPases that promote exosome secretion among 59 Rab GTPases in HeLa cells by performing shRNA screening, i.e., RAB2B, RABSA, RAB9A, RAB27A and RAB27B. Silencing RAB2B, RAB5A, RAB9A, RAB27A and RAB27B could contribute to the reduction of exosome secretion, with a more significant deletion-reduction effect for RAB27A and RAB27B [21]. Both RAB27A and RAB27B are known to play important roles in the process of MVB anchoring to the membrane. However, there are no completely identical roles of these two subtypes in the exosome secretion pathway. Specifically, RAB27A showed a primary association with the anchoring of MVBs on the cell membrane. RAB27A gene knockout might induce an increase in the volume and number of MVBs. However, compared with the control cells, there was no significant change in the intracellular location of MVBs, with an even distribution in the cytoplasm. After RAB27B gene knockout, MVBs gathered significantly around the nucleus, suggesting that MVBs had a relationship with the outward transport of MVBs from the cytoplasm to the cell membrane [49].

AURKB consists of 344 amino acids, including an N-terminal domain, a protein kinase domain and a C-terminal domain. The N-terminal domain of AURKB shares almost no sequence similarity with other Aurora kinase subtypes [50]. Consequently, AURKB has a unique ability to undergo protein–protein interactions with other subtypes. In our study, there was an interaction between phosphorylated AURKB and RAB27B, which increased the protein stability of RAB27B. However, at this stage, it is still unclear whether this is a unique feature of AURKB or whether it is common in other Aurora kinase subtypes. Additionally, there is an absence of relevant studies to confirm that AURKB is a typical transcription factor. Therefore, further exploration is required concerning the mechanism by which AURKB exerts negative regulation of RAB27B at the transcriptional level.

In summary, considering the difference in the phosphorylation level of AURKB between breast cancer cells and wild-type cells, our study confirms the positive correlation between AURKB phosphorylation and drug resistance via both cell and animal experiments. Furthermore, PRKCE upstream regulates the phosphorylation of AURKB and promotes a change in the spatial localization of AURKB from the nucleus to the cytoplasm. The phosphorylation of AURKB may further weaken the negative regulatory role on downstream RAB27B transcription. AURKB can also interact with RAB27B in the cytoplasm to maintain its protein stability, thus increasing exosome secretion and drug efflux in drug-resistant cells. Using shRNA to knock down AURKB expression, using hesperadin to inhibit AURKB activity, mutating the AURKB phosphorylation site, and using siRNA or BIM to inhibit the activity of the upstream AURKB phosphorylation regulatory protein PRKCE, all of which directly or indirectly reduce AURKB phosphorylation, are effective in reversing PTX resistance in cells. Collectively, the findings obtained in our study may provide experimental evidence for the involvement of the PRKCE/AURKB/RAB27B axis in the resistance of breast cancer cells to PTX and offer a potential intervention target for reversing tumor drug resistance.

## Supplementary Information

Below is the link to the electronic supplementary material.

Supplementary file1 (DOCX 1177 KB)

Supplementary file2 (XLSX 22 KB)

Supplementary file3 (XLSX 27 KB)

## References

[1] Schumacher JM, Golden A, Donovan PJ (1998). AIR-2: an Aurora/Ipl1-related protein kinase associated with chromosomes and midbody microtubules is required for polar body extrusion and cytokinesis in *Caenorhabditis elegans* embryos. J Cell Biol.

[2] Mesilaty-Gross S, Reich A, Motro B, Wides R (1999). The Drosophila STAM gene homolog is in a tight gene cluster, and its expression correlates to that of the adjacent gene ial. Gene.

[3] Roghi C, Giet R, Uzbekov R (1998). The Xenopus protein kinase pEg2 associates with the centrosome in a cell cycle-dependent manner, binds to the spindle microtubules and is involved in bipolar mitotic spindle assembly. J Cell Sci.

[4] Nigg EA (2001). Mitotic kinases as regulators of cell division and its checkpoints. Nat Rev Mol Cell Biol.

[5] Adams RR, Wheatley SP, Gouldsworthy AM (2000). INCENP binds the Aurora-related kinase AIRK2 and is required to target it to chromosomes, the central spindle and cleavage furrow. Curr Biol.

[6] Gorbsky GJ (2004). Mitosis: MCAK under the aura of Aurora B. Curr Biol.

[7] Hirota T, Lipp JJ, Toh BH, Peters JM (2005). Histone H3 serine 10 phosphorylation by Aurora B causes HP1 dissociation from heterochromatin. Nature.

[8] Andrews PD, Ovechkina Y, Morrice N (2004). Aurora B regulates MCAK at the mitotic centromere. Dev Cell.

[9] Yasui Y, Urano T, Kawajiri A (2004). Autophosphorylation of a newly identified site of Aurora-B is indispensable for cytokinesis. J Biol Chem.

[10] Parra MT, Gómez R, Viera A (2006). A perikinetochoric ring defined by MCAK and Aurora-B as a novel centromere domain. PLoS Genet.

[11] Sanhaji M, Friel CT, Wordeman L, Louwen F, Yuan J (2011). Mitotic centromere-associated kinesin (MCAK): a potential cancer drug target. Oncotarget.

[12] Honda R, Körner R, Nigg EA (2003). Exploring the functional interactions between Aurora B, INCENP, and survivin in mitosis. Mol Biol Cell.

[13] Ahmed A, Shamsi A, Mohammad T, Hasan GM, Islam A, Hassan MI (2021). Aurora B kinase: a potential drug target for cancer therapy. J Cancer Res Clin Oncol.

[14] Borah NA, Reddy MM (2021). Aurora kinase B inhibition: a potential therapeutic strategy for cancer. Molecules.

[15] Goldenson B, Crispino JD (2015). The aurora kinases in cell cycle and leukemia. Oncogene.

[16] You J, Li Q, Wu C, Kim J, Ottinger M, Howley PM (2009). Regulation of aurora B expression by the bromodomain protein Brd4. Mol Cell Biol.

[17] Bischoff JR, Anderson L, Zhu Y (1998). A homologue of Drosophila aurora kinase is oncogenic and amplified in human colorectal cancers. EMBO J.

[18] Katayama H, Brinkley WR, Sen S (2003). The Aurora kinases: role in cell transformation and tumorigenesis. Cancer Metastasis Rev.

[19] Pike T, Brownlow N, Kjaer S, Carlton J, Parker PJ (2016). PKCɛ switches Aurora B specificity to exit the abscission checkpoint. Nat Commun.

[20] Fukuda M (2013). Rab27 effectors, pleiotropic regulators in secretory pathways. Traffic.

[21] Ostrowski M, Carmo NB, Krumeich S (2010). Rab27a and Rab27b control different steps of the exosome secretion pathway. Nat Cell Biol.

[22] Bray F, Ferlay J, Soerjomataram I, Siegel RL, Torre LA, Jemal A (2018). Global cancer statistics 2018: GLOBOCAN estimates of incidence and mortality worldwide for 36 cancers in 185 countries. CA Cancer J Clin.

[23] Munzone E, Colleoni M (2015). Clinical overview of metronomic chemotherapy in breast cancer. Nat Rev Clin Oncol.

[24] Hart CD, Migliaccio I, Malorni L, Guarducci C, Biganzoli L, Di Leo A (2015). Challenges in the management of advanced, ER-positive, HER2-negative breast cancer. Nat Rev Clin Oncol.

[25] Juliano RL, Ling V (1976). A surface glycoprotein modulating drug permeability in Chinese hamster ovary cell mutants. Biochim Biophys Acta.

[26] Garcia-Mayea Y, Mir C, Masson F, Paciucci R, Lleonart ME (2020). Insights into new mechanisms and models of cancer stem cell multidrug resistance. Semin Cancer Biol.

[27] Garg R, Benedetti LG, Abera MB, Wang H, Abba M, Kazanietz MG (2014). Protein kinase C and cancer: what we know and what we do not. Oncogene.

[28] Chakrabarty S, Huang S (1996). Modulation of chemosensitivity in human colon carcinoma cells by downregulating protein kinase C alpha expression. J Exp Ther Oncol.

[29] Masanek U, Stammler G, Volm M (2002). Modulation of multidrug resistance in human ovarian cancer cell lines by inhibition of P-glycoprotein 170 and PKC isoenzymes with antisense oligonucleotides. J Exp Ther Oncol.

[30] Kim CW, Asai D, Kang JH, Kishimura A, Mori T, Katayama Y (2016). Reversal of efflux of an anticancer drug in human drug-resistant breast cancer cells by inhibition of protein kinase Cα (PKCα) activity. Tumour Biol.

[31] Basu A, Sivaprasad U (2007). Protein kinase Cepsilon makes the life and death decision. Cell Signal.

[32] Meshki J, Caino MC, von Burstin VA, Griner E, Kazanietz MG (2010). Regulation of prostate cancer cell survival by protein kinase Cepsilon involves bad phosphorylation and modulation of the TNFalpha/JNK pathway. J Biol Chem.

[33] Lu D, Huang J, Basu A (2006). Protein kinase Cepsilon activates protein kinase B/Akt via DNA-PK to protect against tumor necrosis factor-alpha-induced cell death. J Biol Chem.

[34] Gorin MA, Pan Q (2009). Protein kinase C epsilon: an oncogene and emerging tumor biomarker. Mol Cancer.

[35] Engers R, Mrzyk S, Springer E (2000). Protein kinase C in human renal cell carcinomas: role in invasion and differential isoenzyme expression. Br J Cancer.

[36] Wang H, Zhan M, Xu SW (2017). miR-218-5p restores sensitivity to gemcitabine through PRKCE/MDR1 axis in gallbladder cancer. Cell Death Dis.

[37] Kumar S, Ingle H, Mishra S (2015). IPS-1 differentially induces TRAIL, BCL2, BIRC3 and PRKCE in type I interferons-dependent and -independent anticancer activity. Cell Death Dis.

[38] Huang B, Fu SJ, Fan WZ (2016). PKCε inhibits isolation and stemness of side population cells via the suppression of ABCB1 transporter and PI3K/Akt, MAPK/ERK signaling in renal cell carcinoma cell line 769P. Cancer Lett.

[39] He SJ, Shu LP, Zhou ZW (2016). Inhibition of Aurora kinases induces apoptosis and autophagy via AURKB/p70S6K/RPL15 axis in human leukemia cells. Cancer Lett.

[40] Lee EC, Frolov A, Li R, Ayala G, Greenberg NM (2006). Targeting Aurora kinases for the treatment of prostate cancer. Cancer Res.

[41] Addepalli MK, Ray KB, Kumar B, Ramnath RL, Chile S, Rao H (2010). RNAi-mediated knockdown of AURKB and EGFR shows enhanced therapeutic efficacy in prostate tumor regression. Gene Ther.

[42] Kurai M, Shiozawa T, Shih HC (2005). Expression of Aurora kinases A and B in normal, hyperplastic, and malignant human endometrium: Aurora B as a predictor for poor prognosis in endometrial carcinoma. Hum Pathol.

[43] Elewaily MI, Elsergany AR (2021). Emerging role of exosomes and exosomal microRNA in cancer: pathophysiology and clinical potential. J Cancer Res Clin Oncol.

[44] Ye X, Bai W, Zhu H (2014). MiR-221 promotes trastuzumab-resistance and metastasis in HER2-positive breast cancers by targeting PTEN. BMB Rep.

[45] Xiao X, Yu S, Li S (2014). Exosomes: decreased sensitivity of lung cancer A549 cells to cisplatin. PLoS ONE.

[46] Maleki S, Jabalee J, Garnis C (2021). The role of extracellular vesicles in mediating resistance to anticancer therapies. Int J Mol Sci.

[47] Lv MM, Zhu XY, Chen WX (2014). Exosomes mediate drug resistance transfer in MCF-7 breast cancer cells and a probable mechanism is delivery of P-glycoprotein. Tumour Biol.

[48] Koch R, Aung T, Vogel D (2016). Nuclear trapping through inhibition of exosomal export by indomethacin increases cytostatic efficacy of doxorubicin and pixantrone. Clin Cancer Res.

[49] Colombo M, Moita C, van Niel G (2013). Analysis of ESCRT functions in exosome biogenesis, composition and secretion highlights the heterogeneity of extracellular vesicles. J Cell Sci.

[50] Carmena M, Earnshaw WC (2003). The cellular geography of aurora kinases. Nat Rev Mol Cell Biol.

