# Supplementary material for: Role of aurora kinase B in regulating resistance to paclitaxel in breast cancer cells
Source: Hum Cell. 2022 Jan 28;35(2):678–93. doi: 10.1007/s13577-022-00675-8 (PMC8866333; doi:10.1007/s13577-022-00675-8)
Supplement: Supplementary file 1 — Supplementary file1 (DOCX 1177 KB) [file 13577_2022_675_MOESM1_ESM.docx]

Supplementary Figures


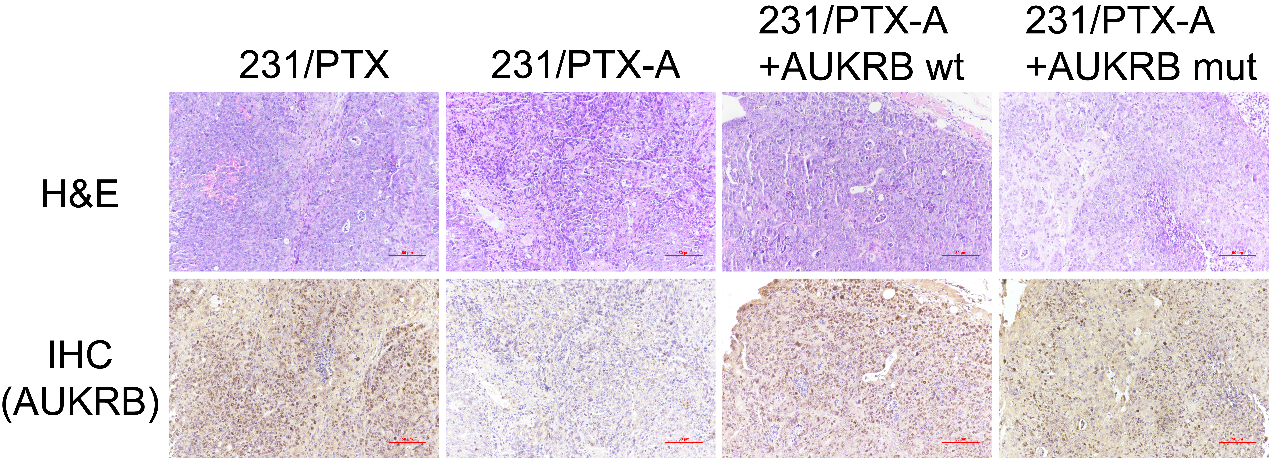


**Fig. S1.** Paraffinembedded tissues of the xenotransplanted tumors were processed for H&E and IHC staining.


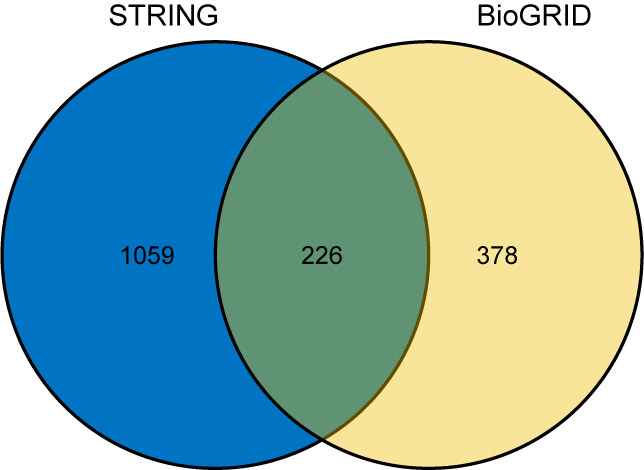


**Fig. S2.** Protein interaction databases STRING (https://string-db.org/) and BioGRID (https://thebiogrid.org/) were used to search for the interacting protein of AURKB. Venn diagram of the potential AURKB interacting proteins
